# Inhalable Antitubercular Therapy Mediated by Locust Bean Gum Microparticles

**DOI:** 10.3390/molecules21060702

**Published:** 2016-05-28

**Authors:** Ana D. Alves, Joana S. Cavaco, Filipa Guerreiro, João P. Lourenço, Ana M. Rosa da Costa, Ana Grenha

**Affiliations:** 1 Center for Biomedical Research (CBMR), Faculty of Sciences and Technology, University of Algarve, 8005-139 Faro, Portugal; anabdiasalves@gmail.com (A.D.A.); joanasofiacavaco@gmail.com (J.S.C.); filiparhg@gmail.com (F.G.); 2 Centre for Marine Sciences (CCMar), University of Algarve, 8005-139 Faro, Portugal; 3 Centro de Química Estrutural (CQE), Instituto Superior Técnico, University of Lisbon, 1049-001 Lisbon, Portugal; jlouren@ualg.pt; 4 Algarve Chemistry Research Center (CIQA) and Department of Chemistry and Pharmacy, Faculty of Sciences and Technology, University of Algarve, 8005-139 Faro, Portugal; amcosta@ualg.pt

**Keywords:** alveolar macrophages, antitubercular drugs, inhalable therapy, locust bean gum, microparticles, spray-drying, tuberculosis therapy

## Abstract

Tuberculosis remains a major global health problem and alternative therapeutic approaches are needed. Considering the high prevalence of lung tuberculosis (80% of cases), the pulmonary delivery of antitubercular drugs in a carrier system capable of reaching the alveoli, being recognised and phagocytosed by alveolar macrophages (mycobacterium hosts), would be a significant improvement to current oral drug regimens. Locust bean gum (LBG) is a polysaccharide composed of galactose and mannose residues, which may favour specific recognition by macrophages and potentiate phagocytosis. LBG microparticles produced by spray-drying are reported herein for the first time, incorporating either isoniazid or rifabutin, first-line antitubercular drugs (association efficiencies >82%). Microparticles have adequate theoretical properties for deep lung delivery (aerodynamic diameters between 1.15 and 1.67 μm). The cytotoxic evaluation in lung epithelial cells (A549 cells) and macrophages (THP-1 cells) revealed a toxic effect from rifabutin-loaded microparticles at the highest concentrations, but we may consider that these were very high comparing with *in vivo* conditions. LBG microparticles further evidenced strong ability to be captured by macrophages (percentage of phagocytosis >94%). Overall, the obtained data indicated the potential of the proposed system for tuberculosis therapy.

## 1. Introduction

Although there is an effective treatment for tuberculosis (TB), the disease remains one of the major health problems worldwide [1]. In 2014, the World Health Organization reported 9.6 million new cases of TB [2], infection caused by inhalation of aerosol particles containing *Mycobacterium tuberculosis* (MTB) bacilli [1]. The inhaled bacilli are phagocytosed by alveolar macrophages, which triggers a series of events that can lead to either control of the infection, *i.e.*, latent TB, or progression to an active form of the disease [1].

Conventional TB therapy involves oral co-administration of several antitubercular drugs over a period of time that may exceed two years in some cases [3]. Severe side effects are frequently reported that, in many cases, lead to therapeutic noncompliance. Therefore, other alternatives are being actively searched to shorten the duration, and perhaps the modality, of treatment [2]. Shortening treatment duration would permit minimising possible side effects in organs such as the liver and kidneys, and avoiding the emergence of resistant TB species. Considering that pulmonary TB represents 80% of cases, the design of antitubercular inhalable formulations is considered an adequate therapeutic approach. Inhalable therapy allows the co-localisation of drugs and pathogens and is thought to enable increased drug concentration in the lungs, along with favoured lung-to-plasma ratio. Overall, the approach is expected to permit reducing doses and frequency of administration, possibly shortening treatment periods, resulting in general therapeutic improvement [3]. Importantly, it has been referred that inhalable TB therapy can either be used to replace oral antibiotherapy, or as add-on therapy [4].

**Figure 1:**
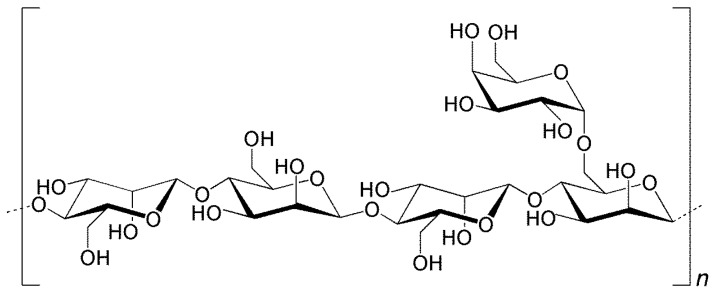
Structure of locust bean gum showing a linear polysaccharide (1–4)-β-linked backbone of mannose units with single (1–6)-α-d-galactose units attached.

Applying this approach requires that the inhalable antitubercular formulations exhibit suitable aerodynamic properties to reach the alveolar zone, where the alveolar macrophages hosting MTB are located [3]. Microparticles have been considered adequate for this end, ensuring not only the protection of the drugs until they reach the site of action, but also providing the necessary aerodynamic characteristics. To reach the alveoli, the aerodynamic diameter should be within 1–3 μm [5], but macrophage uptake is reported to be maximal for particles of 1–2 μm [6,7], thus identifying this size as a target in formulation design. Spray-drying has revealed appropriate to produce polymeric microparticles for inhalation purposes, being a versatile technique that permits tailoring microparticles to the desired characteristics, *i.e.*, size, density and morphology [8]. Different materials have been used in lung drug delivery systems. Polysaccharides are one of the most popular classes, mainly due to their flexible chemical structures, involving many hydroxyl groups available for functionalisation, apart from the high probability of biocompatibility and biodegradability, and availability at a relatively low price [9]. Locust bean gum (LBG) is a neutral polysaccharide of the galactomannan family, with reported pharmaceutical application mainly in tablet production. It is extracted from the seeds of the carob tree (*Ceratonia siliqua*) [10] and consists of a linear chain of (1–4)-linked β-d-mannopyranosyl units with (1–6)-linked side chains of α-d-galactose, in a mannose/galactose ratio of 4:1, as depicted in Figure 1. LBG molecular weight is estimated to be in the range of 50 to 3000 kDa [11]. The application of LBG as a matrix material of microparticles designed to carry and deliver antitubercular drugs can be of great interest owing to the ability of macrophages to recognize and phagocytose with preference materials having structural units such as mannose and galactose [7].

In this work we have prepared spray-dried LBG microparticles for inhalable antitubercular therapy. Microparticles were tailored to exhibit adequate aerodynamic properties to reach the alveolar zone and favour phagocytosis. To the best of our knowledge, LBG microparticles prepared by this method are not described in the literature. The ability to associate two first-line antitubercular drugs, isoniazid (INH) and rifabutin (RFB), was demonstrated. The cytotoxicity of drug formulations, free drugs and unloaded microparticles was tested in relevant respiratory cell lines, *i.e.*, A549 and macrophage-like THP-1 cells, and the ability of macrophages to capture LBG microparticles was established.

## 2. Results and Discussion

### 2.1. Preparation of Locust Bean Gum (LBG) Microparticles by Spray-Drying

Obtaining a polymeric dispersion adequate for spray-drying required a great optimisation of each step, due to the high viscosity of LBG in solution. LBG is described to adopt an extended rod-like conformation in solution, occupying a large volume of gyration, which results in high viscosity [12]. A LBG dispersion at 2% (*w*/*v*) exhibits such a high viscosity that spray-drying is hampered. For the specific LBG polymer being used, Sigma-Aldrich (the provider) reports a viscosity of 2100–3750 cps for a 1% (*w*/*v*) solution, which is much greater than the ideal for an operation with the spray-drying equipment, reported by Buchi (Flawil, Switzerland) to be of 300 cps [13]. The high viscosity of LBG solutions at relatively low concentrations is reported, which is only slightly affected by pH, salts or temperature [14]. Nevertheless, in this work the addition of hydrochloric acid (HCl) 0.1 M was found to provide the adequate viscosity for spray-drying. Additionally, the grinding steps that are described in Section 3.3 and the use of hot water during LBG solubilisation were also very relevant. Several (unsuccessful) attempts were performed to use less concentrated HCl for LBG solubilisation (0.001 M and 0.01 M). The more significant reduction of viscosity in presence of HCl 0.1 M is attributed to a high concentration of H^+^ in solution, which causes a weak protonation of the hydroxyl groups of water and galactomannan molecules, resulting in reduced number of hydrogen bonds. With less bonds being established between water and LBG, and between LBG molecules, there is less expansion of the LBG chain and, therefore, fewer interactions of galactomannans occur, causing a reduction of viscosity [15].

After obtaining a polymeric dispersion with adequate viscosity, LBG microparticles were successfully prepared in a one-step spray-drying process, exhibiting the characteristics that are detailed in the following section.

### 2.2. Association of Drugs and Characterisation of Microparticles

**Figure 2:**
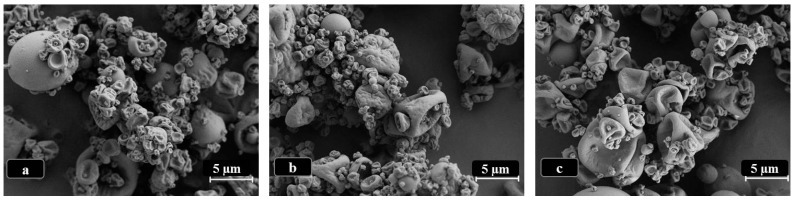
Microphotographs of LBG-based microparticles viewed by scanning electron microscopy. (**a**) Unloaded LBG microparticles; (**b**) LBG:INH = 10:1 (*w*/*w*) microparticles; (**c**) LBG:RFB = 10:0.5 (*w*/*w*) microparticles, the latter representative of formulations containing different amounts of RFB; INH: isoniazid, LBG: locust bean gum, RFB: rifabutin.

LBG microparticles, either in presence or absence of drug, presented a convoluted surface, as depicted in Figure 2. No morphological alterations were perceived after drug incorporation, even when testing different concentrations of RFB, although these photographs are not shown. Many particles presented a spherical or approximately spherical shape, although strongly convoluted, while some others displayed rather irregular morphology.

The observed morphologies are similar to those reported for other spray-dried polysaccharide microparticles including matrixes of hyaluronic acid-hydroxypropyl cellulose [16] and chitosan-gelatin [17]. Nevertheless, spherical shapes have also been reported for many polysaccharide-based spray-dried microparticles [18,19,20], evidencing the important role of the matrix material in this regard. The morphology of spray-dried microparticles is reported to strongly reflect the affinity between the polymer being sprayed and the solvent. In fact, when the droplets of polymer dispersion start drying, a film of polymer is first formed on the surface that might interfere with diffusion of water from the inside out. This leads to an increase of the internal pressure that might reach a critical point where the particle deforms or even disintegrates [21]. The irregular morphology observed in the microparticles of this work may improve the dispersibility and flow properties of dry powders, as surface irregularities will reduce the contact between microparticles and the possibility of establishing van der Waals forces that lead to agglomeration [22]. It is important to highlight that, from an eye observation, the presence of RFB in microparticles seemed to improve dispersibility, an effect that was concentration-dependent, as it was particularly visible for formulations LBG:RFB = 10:1 and 10:0.5 (*w*/*w*). An experimental determination of dispersibility and aerosolisation properties would be very important to reinforce this observation.

Table 1 and Table 2 display the physical and aerodynamic characteristics of LBG microparticles, along with the spray-drying yields. The former were appropriate for the intended application, with aerodynamic diameters varying between 0.89 and 1.83 μm, and Feret’s diameters ranging from 1.10 to 1.50 μm. The latter were considered good, varying between 60% and 70%, thus indicating a low loss of materials and the effectiveness of the technique. The use of the high performance cyclone instead of the conventional cyclone separator strongly contributed to the high yields, an effect that was previously reported [23].

The particle size and the density of inhalable dry powders are prominent factors in the success of the formulations, because they strongly influence the sedimentation and dispersion properties [24]. Among the microparticle formulations developed in this work, no relevant differences were found on the Feret’s diameter, which varied from 1.10 μm to 1.50 μm (Table 1). As expected, the inclusion of drugs had no effect on size, given the relatively low loading. Real, bulk and tap densities were also very similar amongst the formulations, with real density varying within 1.3–1.4 g/cm^3^ (Table 2), very common values for spray-dried powders [18,25]. Bulk densities varied between 0.14 and 0.25 g/cm^3^ and tap densities had slightly increased values in comparison with the former, as a consequence of the method used for the determinations. The real density values, along with Feret’s diameters, resulted in theoretical aerodynamic diameters between 0.9 and 1.8 μm (Table 1). While some microparticles were spherical, the majority was strongly convoluted, as referred above. When microparticles have a clear spherical shape, a shape factor of 1 should be considered [26], but for irregular morphologies the shape factor to be used should be 2 [27]. Therefore, the calculation was performed using a shape factor of either 1 or 2, in order to find an interval of expected aerodynamic diameters [27]. The aerodynamic diameters determined for antitubercular drug-loaded microparticles appear suitable for deep lung deposition upon inhalation.

Obtaining efficient association of antitubercular drugs was one of the objectives of this work. Spray-drying is a technique usually providing high association efficiencies [28], which was reinforced in this study, where encapsulation efficiencies up to 100% were observed. Association efficiency and loading capacity of LBG microparticles are displayed in Table 3.

INH and RFB were associated with approximately equal efficiency, demonstrating that the process was independent of the aqueous solubility of the drugs (125 mg/mL for INH and 0.19 mg/mL for RFB). Owing to its hydrophilic nature, INH was easily dissolved in the LBG solution and, thus, the association efficiency of 89% was not surprising, being similar to that reported for other INH-loaded spray-dried microparticles [29,30]. Such association efficiency resulted in a loading capacity around 9%. Contrary to INH, RFB is a lipophilic drug and its association to the microparticles required an optimisation of the solubilisation conditions. The presence of HCl in the dissolving medium, included to decrease the viscosity of LBG solution (Section 2.1), induced the protonation of the drug that allows an increase of the solubility in water and the adequate incorporation in the LBG dispersion. The concomitant trituration of LBG and RFB powders further contributed to the successful association of RFB, demonstrating the importance of improving solid dispersions. RFB-loaded microparticles were produced with different LBG:RFB mass ratios (10:1, 10:0.5; 10:0.2) after verifying the strong cell toxicity induced by RFB. This was not observed for INH, justifying that only the formulation LBG:INH (10:1, *w*/*w*) was produced. As shown in Table 3, RFB association efficiency was very high in all cases (86%–100%). The highest association efficiency (100%, *p* < 0.05) was observed for the formulation with the highest theoretical loading, that is, the formulation LBG:RFB = 10:1 (*w*/*w*), which also showed the highest loading capacity (10%). Although the association efficiency of the other two formulations was not significantly different (86%–92%), the loading capacity of LBG:RFB = 10:0.5 microparticles (4%) was significantly higher than that of LBG:RFB 10:0.2 microparticles (2%) because the former had a higher amount initially added of RFB (5% *vs.* 2%). Overall, the loading capacities are in line with the theoretical loadings initially established for the preparation of microparticles, owing to the high association efficiencies.

### 2.3. Cristallinity of LBG-Based Microparticles

X-ray diffraction (XRD) was used to assess LBG and unloaded LBG microparticles, drugs before and after spray-drying, and drug-loaded LBG microparticles. Diffractograms resulting from drug assessment are presented in Figures S1 and S2 (Supplementary Materials), for INH and RFB, respectively. INH was prepared as aqueous solution and RFB as acidic solution. INH (commercial sample) displays a XRD pattern with sharp and intense peaks, denoting a highly crystalline structure. The pattern matches that described in the literature [31] and no other peaks than those belonging to the INH crystalline phase were found. The pattern recorded after the spray-drying process indicates the presence of crystalline INH. However, the different relative peak intensities, when compared with parent INH (Figure S1), suggest a different preferential orientation probably caused by drug recrystallization. The XRD pattern of RFB recorded before the spray-drying process (Figure S2) shows the typical diffraction peaks of the crystalline drug [32]. These peaks completely vanish after the spray-drying process, indicating substantial reduction of crystallinity. In this case, the spray-drying process seems to promote the formation of amorphous RFB, although the presence of small-sized crystals of RBF (not detectable by XRD), formed either by recrystallization or dehydration, cannot be discarded.

**Figure 3:**
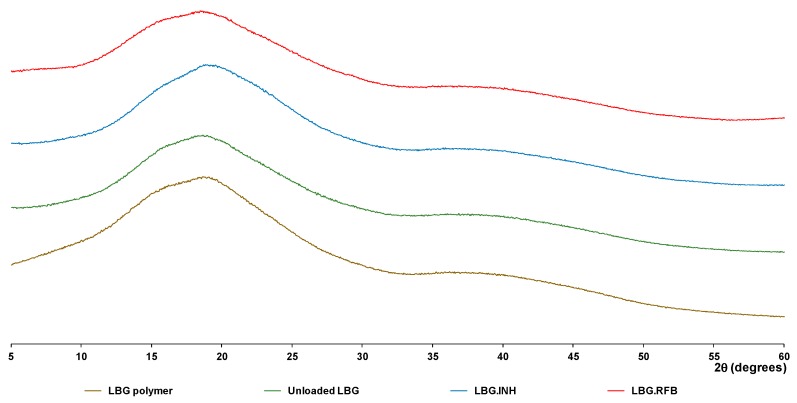
XRD diffractograms of locust bean gum (LBG) raw material and spray-dried formulations: LBG raw material (**brown** line), unloaded LBG microparticles (**green** line), LBG:INH 10:1 microparticles (**blue** line), LBG:RFB 10:1 microparticles (**red** line); INH: isoniazid; LBG: locust bean gum; RFB: rifabutin.

Diffractograms of LBG, unloaded LBG microparticles and drug-loaded LBG microparticles are presented in Figure 3. For the formulation containing RFB, the diffractogram of LBG:RFB 10:1 is presented, which is considered representative of the other ratios.

Locust bean gum does not show diffraction peaks, in agreement with its non-crystalline nature. The diffraction patterns obtained after and before spray-drying are similar to each other, and are also similar to those described in the literature [33]. Spray-dried INH retains high crystallinity when compared with RFB, as shown in Figures S1 and S2 (Supplementary Materials). Therefore, at least the most intense diffraction peaks from INH were expected to be observed in microparticle formulations (these peaks were clearly visible in the diffractogram of a physical mixture 10:1 of LBG and INH—Figure S3). Nevertheless, in the diffractogram of LBG:INH microparticles no diffraction peaks were identified. The patterns are identical among themselves and also similar to that of the polymer. This observation is common to other spray-dried microparticles [4,30], where INH peaks disappear in INH-loaded microspheres, only peaks from the matrix material being observed. This absence of INH peaks might be due to the fact that very small crystals are formed after INH recrystallization on the matrix of LBG, which are below the detection limit of the equipment [34]. It may also be justified by the rapid process of drying occurring in spray-drying, which affects crystal rearrangement, leading to the formation of an amorphous phase [28]. On the other hand, high inlet temperatures used in the process have also been referred in the literature as possibly affecting the final structure [35]. In this regard, the production of INH-loaded microparticles was also performed at 120 °C (instead of 160 °C), but no differences in the crystallinity were found (data not shown), indicating an absence of the effect of inlet temperature in this case. The diffractogram of LBG:RFB does not show any additional diffraction peak when compared with that of unloaded LBG. This was somewhat expected, because the spray-drying process itself promotes the loss of crystallinity of RFB, as described above.

### 2.4. In Vitro Drug Release

The medium selected to evaluate the release of the drugs consisted of phosphate buffered saline (PBS) pH 7.4 added of 1% Tween 80^®^. The latter, apart from contributing to the resemblance with the surfactant present in the lung lining fluid, is essential to enable the dissolution of RFB, which is not soluble in PBS. The presence of Tween 80^®^ at the used concentration was reported not to affect spectrophotometric measurements at the considered wavelength [36].

**Figure 4:**
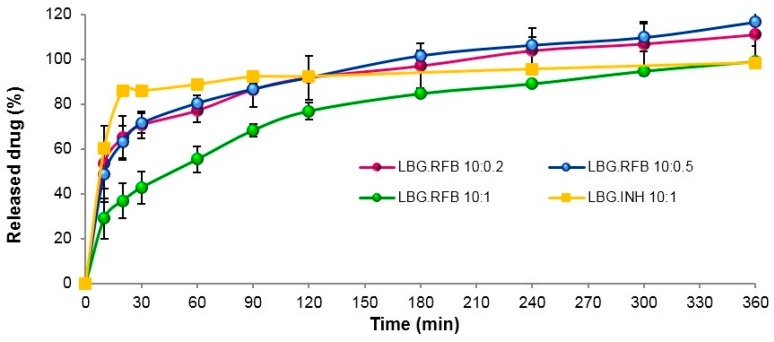
*In vitro* release of isoniazid (INH) from LBG:INH (10:1, *w*/*w*) microparticles and of rifabutin (RFB) from LBG:RFB microparticles at mass ratios 10:0.2, 10:0.5 and 10:1, in PBS pH 7.4.Tween 80^®^ (LBG: locust bean gum; mean ± SD, *n* = 3).

The release profile of both INH and RFB from the respective microparticle formulations is plotted in Figure 4. INH-loaded microparticles have the fastest release (86% in 20 min), which was expected owing to the high solubility of INH. In general, this profile is identical to that obtained from INH-loaded polylactic acid microparticles in PBS pH 7.4 [29].

The release of RFB was somewhat slower at initial time points, particularly for microparticles with the highest amount of the drug (LBG:RFB 10:1, *w*/*w*), although it still corresponds to a rapid release profile. This is the formulation permitting the most direct comparison with INH-loaded microparticles, because the same amount of drug is associated. In the same interval where LBG:INH microparticles released 86% (20 min), LBG:RFB 10:1 (*w*/*w*) microparticles released 37% (*p* < 0.05). This difference is certainly a consequence of the different water solubility of each drug. As said, the other two formulations containing RFB (LBG:RFB 10:0.5 and 10:0.2, *w*/*w*) comparatively showed a faster release (63% in the same period, *p* < 0.05), with a similar release pattern during the whole assay. Additionally, these reached 100% release at 240 min, while at that time LBG:RFB 10:1 (*w*/*w*) microparticles reached only 80%.

In spite of the slight differences detailed above, the release was generally rapid for all formulations, which is possibly due to two factors. First, the high specific surface area of these convoluted microparticles, which provides improved contact with the release medium. Moreover, a second reason can be the apparent absence or very low crystallinity pattern of both INH- and RFB-loaded microparticles that potentiates a rapid dissolution in the release medium. Nevertheless, a certain sustained release effect is observed for RFB-loaded microparticles, although it lasts for a short time, which is attributed to the gelling ability of LBG along with the lower aqueous solubility of RFB. When the assay starts and microparticles initiate the contact with the aqueous medium, the polymer matrix gradually begins to hydrate from periphery to centre, forming a gelatinous swollen mass, which progressively allows the release of drugs into the medium [37]. It is also important to stress that, although drug release is fast and it could be thought to occur before microparticle internalization by cells, real *in vivo* conditions of the lung are not being simulated. The alveolar zone has a much lower amount of liquid comparing with that involved in the assay and, therefore, the release rate is expected to be slower than that reported. Therefore, this experimental setup is more useful and accurate at providing a comparison between formulations than at predicting *in vivo* occurrences.

### 2.5. Cytotoxic Evaluation

The cytotoxic evaluation of LBG microparticles was performed in two cell lines of the alveolar environment, epithelial A549 cells and macrophage-differentiated THP-1 cells. The differentiation is performed with phorbol 12-myristate 13-acetate (PMA) and is reported to induce a phenotype having characteristics of alveolar macrophages [38]. Evidence of this process is presented as Supplementary Materials (Figure S4). Different *in vitro* tests were employed, allowing the evaluation of the cell metabolic activity (thiazolyl blue tetrazolium bromide (MTT) assay) and membrane integrity (lactate dehydrogenase (LDH) release assay) after exposure to drug formulations.

#### 2.5.1. Evaluation of Metabolic Activity

Free drugs (INH and RFB), raw material (LBG) as obtained commercially, unloaded LBG microparticles and drug-loaded LBG microparticles were incubated with cultures of the two cell lines. A shorter (3 h) and a more prolonged period of exposure (24 h) were tested, and three concentrations of materials were assessed (0.1, 0.5 and 1 mg/mL). Considering that the theoretical drug loading of microparticles was 10%, free drugs were evaluated at 10x lower concentrations comparing with the other materials (0.01, 0.05 and 0.1 mg/mL). In all cases samples were presented as solutions/suspensions prepared in pre-warmed cell culture medium (CCM). For the discussion of results, it was considered that a material has cytotoxic potential when cell viability after exposure to the material decreases below 70% (indicated with a dashed line in all figures), as designated by the ISO 10993-5 [39].

**Figure 5:**
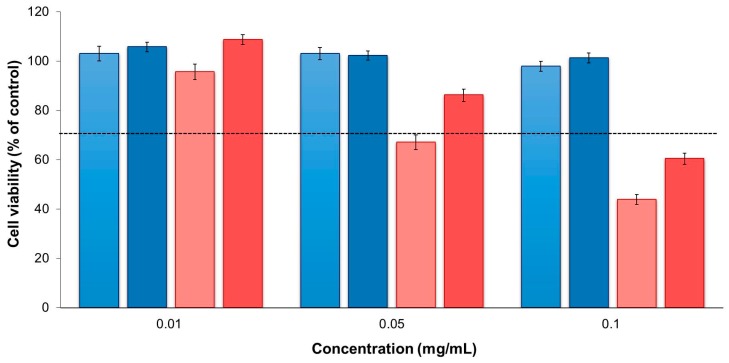
A549 (**lighter** colour) and macrophage-differentiated THP-1 (**darker** colour) cell viabilities after 24 h of exposure to free drugs, isoniazid (**blue**) and rifabutin (**red**). Results are expressed as mean ± SEM (*n* = 3, six replicates per experiment at each concentration). Dashed line represents 70% cell viability.

The exposure of cells to the free drugs revealed two important aspects. INH did not show any detrimental effect on cell viability, which remained around 90%–100% in all cases, irrespective of the cell line, tested concentration and time of exposure (Figure S5). On the contrary, time- and concentration-dependent effects were observed for RFB (*p* < 0.05). After 3 h of exposure to this drug, cell viabilities remained above 80% (Figure S6), but a strong decrease was observed after 24 h (Figure 5). This decrease was particularly noticeable for the highest concentration tested (0.1 mg/mL). There was also a trend indicating lower viabilities obtained in A549 cells, suggesting higher sensitivity of this cell line comparing with differentiated THP-1 cells upon contact with the free drugs.

LBG and LBG-based microparticles were evaluated separately, in order to disclose an effect from the carrier structure [40]. Testing LBG is not only a relevant control of the work, but also contributes to the state of the art, as no similar evaluation is, to our knowledge, described in the literature.

**Figure 6:**
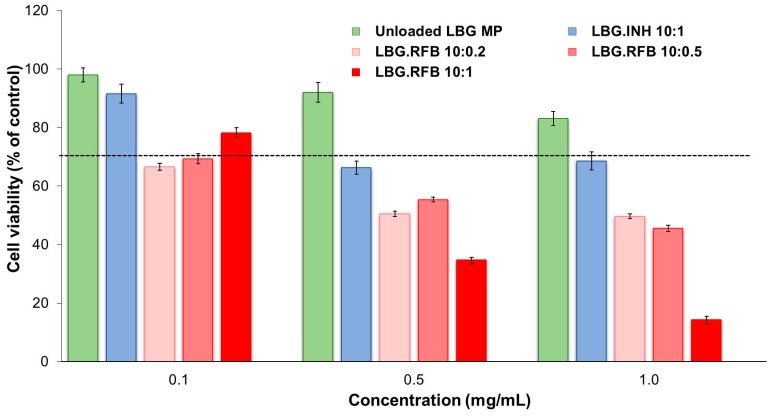
A549 cell viabilities after 24 h of exposure to LBG-based microparticle formulations. Results are expressed as mean ± SEM (*n* = 3, six replicates per experiment at each concentration). Dashed line represents 70% cell viability (INH: isoniazid, LBG: locust bean gum, RFB: rifabutin).

Again, while no overt toxicity was found in THP-1 cells (cell viability >85% in all conditions), A549 cells demonstrated to be more sensitive, showing 40% cell viability after 24 h of exposure independently of the concentration (Figure S7). Interestingly, this detrimental effect completely reverted after spray-drying, as unloaded LBG microparticles exhibited a very mild effect even after 24 h (A549 cell viability >75% in all cases, Figure 6).

The described effect of LBG as raw material after the contact with A549 cells could be attributed to either of two reasons: (i) the occurrence of a partial hydrolysis of commercial LBG in the acidic solution either prior to spray-drying or potentiated by the heat and shear forces of the process, leading to lower molecular weight polymer chains; or (ii) a slower solubilisation of the microparticles when compared to the commercial powder, which delays the increase of viscosity in the solution to be tested. This translates directly to an effect at the level of the viscosity of dispersions prepared at the same concentration using LBG commercial powder and unloaded LBG microparticles. As the assay is performed by incubating a dispersion of polymer/microparticles with cells, higher viscosity of the dispersion possibly makes gaseous exchanges between cells, medium and air a difficult task. This could lead to higher cell death. In fact, the dispersion obtained from LBG microparticles was perceived as more fluid than that prepared from commercial LBG polymer.

**Figure 7:**
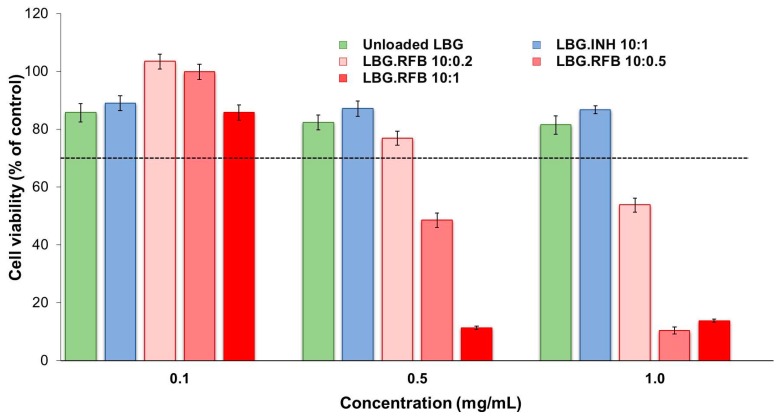
THP-1 cell viabilities after 24 h of exposure to LBG-based microparticle formulations. Results are expressed as mean ± SEM (*n* = 3, six replicates per experiment at each concentration). Dashed line represents 70% cell viability (INH: isoniazid, LBG: locust bean gum, RFB: rifabutin).

Concerning macrophage-like THP-1 cells, no relevant variation of cell viability was observed upon exposure to either a dispersion of commercial LBG (Figure S7) or unloaded LBG microparticles (Figure 7). This observation is again in line with a higher sensitivity of A549 cells to the materials, comparing with THP-1 cells.

The results of the cytotoxic evaluation of drug-loaded LBG microparticles are presented in Figure 6 and Figure 7, representing the 24 h exposure of A549 and macrophage-differentiated THP-1 cells, respectively. The results obtained after a short contact time (3 h) are available as Supplementary Materials (Figures S8 and S9) and show similar tendencies, although in some cases the determined cell viabilities were higher than those at 24 h, indicating a time-dependent effect.

The general trend indicates that both cell lines responded to the presence of drug-loaded formulations according to a similar pattern, that is, INH-loaded microparticles induced low effect on cell viability and RFB-loaded microparticles elicited considerable cytotoxicity. More specifically, INH-loaded microparticles induced cell viabilities above 70% in all tested conditions (exposure times, concentrations and cell lines) with the exception of the 24 h exposure of A549 cells to concentrations of 0.5 and 1 mg/mL, which resulted in viabilities of 66%–68% (Figure 6). It was also observed an absence of concentration-dependent effect for this formulation, at 3 h and 24 h. The only exception occurs in A549 cells after 24 h exposure, where 0.1 mg/mL of INH-loaded microparticles induced 92% cell viability, contrasting with 66%–68% when testing 0.5 and 1 mg/mL (*p* < 0.05). Worth mentioning is the fact that responses to INH-loaded microparticles were very similar to those generated by unloaded LBG microparticles in both cell lines, giving a clear indication on the absence of toxicity of INH itself. No overt cytotoxic effect was, thus, considered to occur for INH-loaded microparticles. These observations are in line with the literature, as the IC_50_ of INH is reported as 1000 mg/mL in alveolar macrophages (isolated from albino rats) [41]. The referred study was performed in primary cells, thus different from those used in this study, but they mimic *in vivo* conditions in a closer way and are supposedly more sensitive than established cell lines [38].

The response to RFB-loaded microparticles contrasted well with the previous results. After a contact of 3 h, a reduction of cell viability in both lines to values around 40%–50% and even 20% (THP-1 cells), particularly for the concentrations of 0.5 and 1.0 mg/mL (Figures S8 and S9), was observed. After 24 h, cell viabilities were generally not very different from those at 3 h, with the relevant exception corresponding to the highest concentration of microparticles (1 mg/mL), namely for LBG:RFB = 10:1 (*w*/*w*) microparticles (Figure 6 and Figure 7). In these conditions, cell viabilities of 10%–15% were determined (*p* < 0.05). In A549 cells, a time-dependent effect was observed as a whole (*p* < 0.05), although it was more pronounced for microparticles LBG:RFB 10:1 (*w*/*w*). A significant difference was also perceived between the various RFB-loaded microparticles (*p* < 0.05), in the order LBG:RFB = 10:1 > 10:0.5~10:0.2, reinforcing that the cytotoxic effect is due to RFB. THP-1 cells also revealed a time-dependent effect (*p* < 0.05), which was particularly visible for microparticles LBG:RFB = 10:1 and 10:0.5 (*w*/*w*) and for the two highest concentrations tested (0.5 and 1 mg/mL). A dose-dependent effect was also visible for RFB-loaded microparticles (*p* < 0.05), which was more pronounced than that observed for A549 cells.

The above mentioned trend, indicating higher susceptibility of A549 cells when compared with THP-1 cells, was thus not followed when testing microparticles, where relatively similar responses are observed between both cell lines. The literature reports opposite effects, either demonstrating higher resistance of differentiated THP-1 cells [42,43] or establishing lower susceptibility for A549 cells [44], clearly indicating that the generated responses are strongly dependent on the assessed materials. What is clearly seen in our study is that there was a difference of susceptibilities when testing free drugs and polymer, which are exposed as solutions, comparing with microparticles. This different outcome is probably related with specific endocytic–exocytic mechanisms of phagocytic and non-phagocytic cells, along with the specialized physiological role of each particular cell type. THP-1 cells are phagocytes with significant endocytic and exocytic activity and natural ability to uptake particulate matter [43]. Therefore, they possibly respond with higher intensity to the ingestion of particulates [44]. On the contrary, epithelial cells possibly have more intimate contact with dissolved solutes than with the corresponding particulates.

#### 2.5.2. Evaluation of Cell Membrane Integrity

As a complementary study, cell membrane integrity was evaluated upon exposure to the different materials. Taking into account the results obtained in the MTT assay, released LDH was determined after 24 h of exposure to the highest concentration tested, 1 mg/mL. For RFB-loaded microparticles, the concentration of 0.5 mg/mL was also tested. RFB was tested as free drug at the concentrations of 0.05 and 0.1 mg/mL while free INH was only tested at 0.1 mg/mL.

**Figure 8:**
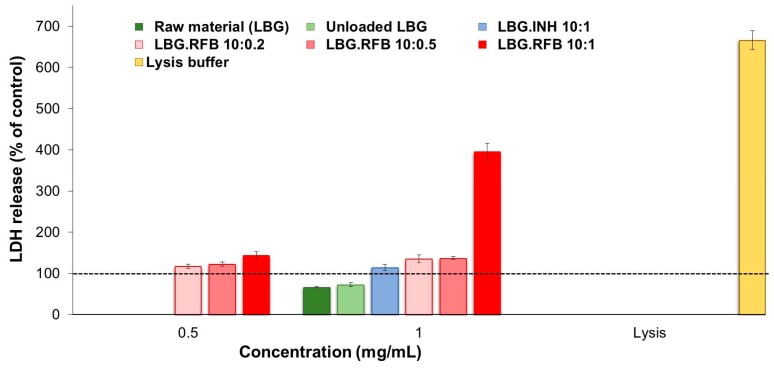
LDH released from A549 cells after 24 h exposure to LBG polymer, LBG microparticles and lysis buffer. Amount of LDH released from cells incubated with cell culture medium is assumed as 100% (dashed line). Results are expressed as mean ± SEM (*n* = 3, six replicates per experiment at each concentration; INH: isoniazid; LBG: locust bean gum; RFB: rifabutin).

**Figure 9:**
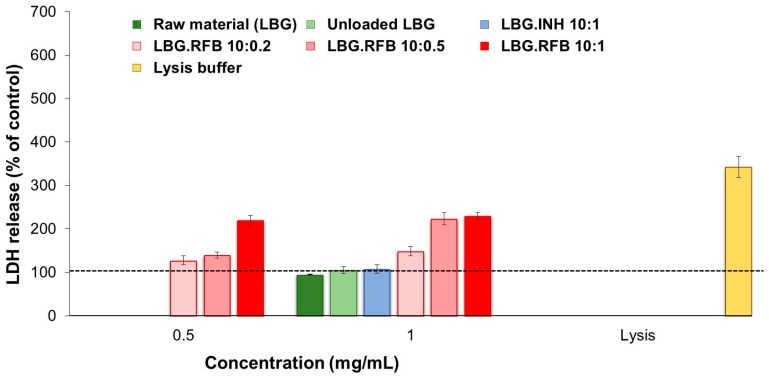
LDH released from macrophage-differentiated THP-1 cells after 24 h exposure to LBG polymer, LBG microparticles and lysis buffer. Amount of LDH released from cells incubated with cell culture medium is assumed as 100% (dashed line). Results are expressed as mean ± SEM (*n* = 3, six replicates per experiment at each concentration; INH: isoniazid; LBG: locust bean gum. RFB: rifabutin).

The results of free drugs are available as Supplementary Materials (Figure S10), being in agreement with those of the MTT assay for both cell lines and reinforcing the observations of RFB cytotoxicity. While the contact with INH did not increase significantly the release of LDH, RFB induced an increase to 150%–170%, which was particularly noticeable for the highest concentration of the drug (0.1 mg/mL). The assessment of the effect of LBG microparticles was also in line with MTT results. Figure 8 and Figure 9 show the observations performed in A549 and macrophage-like THP-1 cells, respectively. The first remarkable observation was related with the effect of LBG as raw material. The MTT assay had shown very different outcomes between the raw material and the unloaded microparticles. However, these differences did not appear at the level of LDH release, as the amount of released enzyme was similar in both cases, for both cell lines. Therefore, although cell death was observed after the MTT assay, it was not related with events at the level of membrane integrity. Additionally, it is important to mention that the amount of enzyme that was released was comparable or even lower than that observed for the control (cells incubated with culture medium).

As such, it was also verified that the amount of released LDH induced by the contact with INH-loaded microparticles was, in both cell lines, similar to that induced by unloaded microparticles, raw material and the control, again evidencing an absence of toxicity of INH.

The observations were very different for RFB-loaded microparticles, justifying the assessment of two concentrations of these microparticles. In A549 cells, the three RFB-loaded microparticles induced similar effect when tested at the lower concentration (0.5 mg/mL), with only a slight increase in LDH release to 110%–140%. Interestingly, the cells responded in a similar manner to a doubled concentration (1 mg/mL) of LBG:RFB 10:0.5 and 10:0.2 (*w*/*w*) microparticles, with no significant alterations in LDH release. However, LBG:RFB 10:1 (*w*/*w*) microparticles elicited a stronger increase in LDH release (*p* < 0.05) to approximately 400%, indicating a clear concentration-dependent effect. The exposure to the lysis buffer, indicating the highest LDH amount possibly released, induced 666%.

The trend was approximately similar in THP-1 cells. LBG:RFB 10:0.2 (*w*/*w*) microparticles induced 125%–150% LDH release at both concentrations tested. A clear concentration-dependent effect was observed for the formulation 10:0.5 (*w*/*w*), with LDH release increasing from 139% to 223% with the increase of concentration (*p* < 0.05). LBG:RFB 10:1 (*w*/*w*) microparticles induced similar release at both concentrations (220%–230%). The lysis buffer induced a value around 350%.

The general observation from the whole set of results of cytotoxicity is that an increased toxic effect is seen when RFB is included in the microparticles. The high *in vivo* toxicity of RFB is well reported [45]. Although the mechanism is still not well established, the cytotoxic behavior might be due in part to the lipophilic character of RFB. It presents a high membrane lipid tropism, resulting in high penetration into the cells, which can impose increased toxicity [46].

Notwithstanding the determinations that were made in this study, there is the expectation that the toxicity does not translate to such a severe level *in vivo*. This belief is based on the assumption of a relatively even distribution of the dry powder in the alveolar zone upon inhalation. The highest dose tested in the described assays (1 mg/mL) corresponds to 303.03 μg/cm^2^. The area of the epithelial surface of alveolar zone is about 70 m^2^ (700,000 cm^2^) [47]. If an even distribution of microparticles is assumed and it is considered that a nominal proportion (e.g., a third) of an inhaled dose (considering the approximate 160 mg of powder delivered by the TOBI^®^ Podhaler in one dose) deposits in the alveolar region, the dose estimated across this area is 0.08 μg/cm^2^. This dose is remarkably lower than the 303.03 μg/cm^2^ used in our study. However, one should bear in mind that the lung of tuberculosis patients possibly has a much lower area, apart from certainly being variable among patients. Still, even if only 10% of the alveolar area is considered functional (7 m^2^), the dose will be 0.76 μg/cm^2^, very far from that used in this study. Taking this into account, the effects will be much closer to those of the lower dose of 0.1 mg/mL than to those of the higher dose or even the 0.5 mg/mL. Unfortunately, we could not meet the conditions permitting weighing such a low amount of dry powder that could resemble in a better way the *in vivo* conditions. Another reason contributing to a possible decrease of *in vivo* toxicological effects associated with the microparticles is their capacity to form complexes with polar heads of groups of the phospholipids present in the pulmonary surfactant, allowing reaching high concentrations without damaging the epithelium [48].

Several other issues related to the toxicity need to be discussed and addressed experimentally in the near future in order to verify the real possibilities of using LBG microparticles for the proposed application. LBG has been clearly referred as biodegradable when administered orally, owing to the presence of β-mannosidase in the human intestine [49]. Importantly, the enzyme has also been detected in the lung, although in lower concentration comparing with other organs [50]. As long-term dosing is needed in the application focused in this work, it is very important to ensure the biodegradability of the microparticles and, although the presence of β-mannosidase is a promising indication, more studies are needed in this regard. In parallel, it is important to unveil the immunological effects of these microparticles, as LBG is a novel polymer in lung delivery and polysaccharides are particularly susceptible in this regard. We are currently performing *in vivo* assays to verify the immunological response to the administration of LBG microparticles by inhalation.

### 2.6. Preliminary Evaluation of Macrophage Ability to Uptake LBG Microparticles

The ability of LBG microparticles to be taken up by alveolar macrophages is crucial to allow the co-localisation of microparticles with the tuberculosis pathogenic agent. Microparticle uptake was evaluated in two macrophage cell lines (human macrophage-differentiated THP-1 cells and rat alveolar macrophages NR8383). The assay was performed by flow cytometry, requiring the use of fluorescently-labelled LBG microparticles. LBG was labelled with fluorescein (fluorescein sodium salt was activated by *N*-(3-dimethylaminopropyl)-*N*′-ethylcarbodiimide hydrochloride (EDAC), at pH 4, and reacted with the nucleophilic hydroxyl groups of LBG, resulting in a fluorescent polymer). This was then used to produce microparticles specifically for this assay, with Feret’s diameter similar to that of unloaded LBG microparticles. In the analysis, cells exhibiting fluorescence were assumed to have phagocytosed microparticles. Two different doses were tested (50 and 220 μg/cm^2^) and the contact with the cells was allowed for 2 h. Phagocytosis is a fast process, usually 50%–75% of the particles are phagocytosed in 2–3 h, 90% or more in 10 h, and nearly 100% at 24 h after particle deposition [51]. The time of 2 h, selected to perform the assay, is deemed adequate for the occurrence of phagocytosis and was also used by other authors [52].

**Figure 10:**
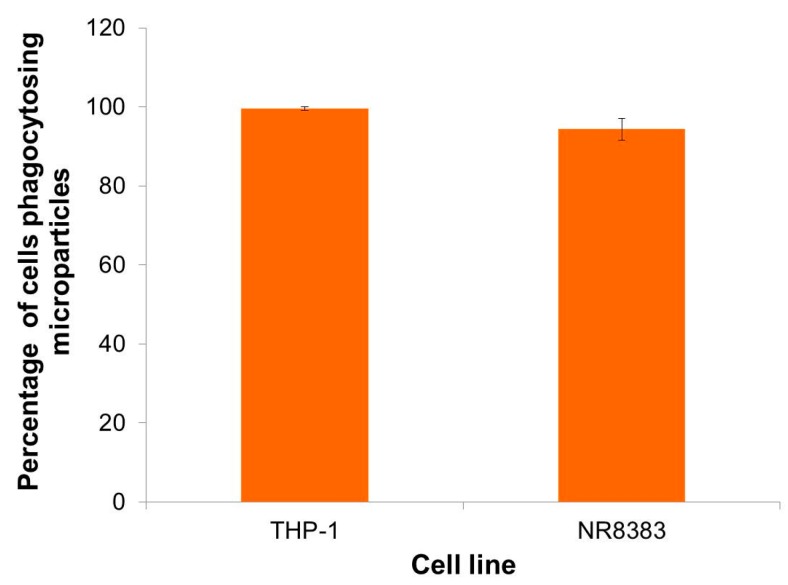
Uptake of fluorescently-labelled locust bean gum (LBG) microparticles by macrophage-differentiated THP-1 cells and NR8383 cells upon exposure to 50 μg/cm^2^, for a period of two hours. Results are expressed as mean ± SEM (*n* ≥ 3).

Macrophage-differentiated THP-1 cells were exposed to the two microparticle concentrations referred above. The percentage of macrophages taking up LBG microparticles was very high in both cases (99.6 ± 0.2% for 220 μg/cm^2^ and 99.5 ± 0.4% for 50 μg/cm^2^). This evidenced an absence of effect of concentration and suggests a high affinity of macrophages for LBG microparticles. Using such different concentrations has been reported to permit the observation of dose-dependent effects on phagocytosis of microparticles of other materials [53], but this did not occur for LBG. Considering these results, NR8383 cells were exposed only to the lowest concentration of microparticles and results are depicted in Figure 10.

As indicated above for differentiated THP-1 cells, rat alveolar macrophages also showed high affinity for LBG microparticles, as 94.4% of the macrophages of the population exhibited fluorescent signal, indicating the occurrence of phagocytosis. No significant differences were found between the uptake by both cells. The graphics depicting the analysis of the populations corresponding to cells of each line not exposed to fluorescently-labelled LBG microparticles (control, incubated with CCM) and cells exposed to fluorescently labelled microparticles, are available as Supplementary Materials (Figure S11). Cells not exposed to microparticles show a certain degree of auto-fluorescence, while cells exposed to fluorescent microparticles evidence a stronger increase of the fluorescence signal.

A preliminary demonstration of the affinity of macrophages for these microparticles was thus provided herein. Nevertheless, as LBG has a chemical structure bearing mannose and galactose units, which are reported to be recognised by macrophage surface receptors [54], a more rigorous determination of its ability for preferential macrophage capture would be provided by a comparison with a material devoid of the recognisable units.

## 3. Experimental Section

### 3.1. Materials

LBG (*M_w_* 860 *k*Da [55]), INH, Tween 80^®^, PBS tablets pH 7.4, Dulbecco’s modified Eagle’s medium (DMEM), l-glutamine solution (200 mM), non-essential amino acids solution and penicillin/streptomycin (10,000 units/mL, 10,000 g/mL), trypsin-EDTA solution (2.5 g/L trypsin, 0.5 g/L EDTA), trypan blue solution (0.4%), PMA, MTT, EDAC, sodium dodecyl sulfate (SDS), dimethylformamide (DMF), dimethyl sulfoxide (DMSO), HCl and LDH kit were purchased from Sigma-Aldrich (Munich, Germany). RFB was supplied by Chemos (Regenstauf, Germany) and fetal bovine serum (FBS) by Gibco (Life Technologies, Waltham, MA, USA). RPMI 1640 and Ham’s F12 media were obtained from Lonza Group AG (Basel, Switzerland). Ultrapure water (MilliQ, Millipore, Feltham, UK) was used throughout. All other chemicals were reagent grade.

### 3.2. Cell Lines

A549 cells (human alveolar epithelium) and NR8383 cells (rat alveolar macrophages) were obtained from the American Type Culture Collection (ATCC, Middlesex, UK) and used in passages 27–37 and 9–18, respectively. THP-1 cells (human monocytes) were obtained from the Leibniz-Institut DSMZ (Braunschweig, Germany) and used in passages 10–20. Cell cultures were grown in humidified 5% CO_2_/95% atmospheric air incubator at 37 °C (HerAcell 150, Heraeus, Hanau, Germany). Cell culture medium (CCM) for A549 cells was DMEM supplemented with 10% (*v*/*v*) FBS, 1% (*v*/*v*) l-glutamine solution, 1% (*v*/*v*) non-essential amino acids solution and 1% (*v*/*v*) penicillin/streptomycin. For NR8383 cells, CCM consisted of Ham’s F12 supplemented with 15% (*v*/*v*) FBS, 1% (*v*/*v*) l-glutamine and 1% (*v*/*v*) penicillin/streptomycin, while THP-1 cells were grown in RPMI 1640 medium supplemented with 10% (*v*/*v*) FBS, 1% (*v*/*v*) l-glutamine and 1% (*v*/*v*) penicillin/streptomycin.

THP-1 cells were grown in suspension and cell culture was maintained between 0.2 × 10^6^ and 0.8 × 10^6^ cells/mL. When reaching this higher concentration, cells were sub cultivated in new passage at the concentration of 0.2 × 10^6^ cells/mL. Differentiation of THP-1 monocytes to provide the macrophage phenotype was performed using PMA (0.2 × 10^6^ cells/mL, 50 nM, 48 h exposure), after which the medium was replaced by fresh medium without PMA for 24 h before the experiments. NR8383 cells grow in mixed culture (half population keeps adherent and half suspended). Adherent cells were those used to perform the assays described below and their harvesting was made by scraping.

### 3.3. Preparation of Locust Bean Gum Microparticles by Spray-Drying

**Figure 11:**
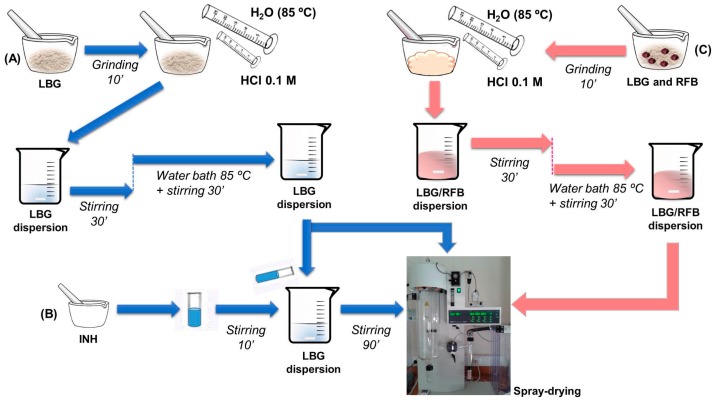
Schematic representation of experimental steps involved in the preparation of locust bean gum (LBG) microparticles, unloaded or loaded with antitubercular drugs. (**A**) Method of preparation of LBG dispersion is the basis of the process; (**B**) Isoniazid (INH) was previously triturated and dissolved in water, being subsequently added to the LBG dispersion for the preparation of LBG.INH microparticles; (**C**) Rifabutin (RFB) was added to the LBG powder, and both were triturated, the rest of protocol being the same applied for LBG only.

As depicted in Figure 11, different procedures were necessary to prepare the dispersions to obtain unloaded and drug-loaded microparticles.

The preparation of unloaded LBG microparticles (Figure 11—route A) involved grinding LBG in a glass mortar for 10 min, after which 5 mL HCl 0.1 M were slowly added and grinding continued until complete mixture of powder and HCl solution was obtained. This was followed by the addition of purified water previously heated to 85 °C, up to a final volume of 50 mL. The concentration of LBG in the final dispersion was 2% (*w*/*v*). The dispersion was maintained under magnetic stirring for 30 min and subsequently placed on a water bath at 85 °C under slow stirring for an additional 30 min. At the end, the dispersion was kept under stirring at room temperature overnight, until the moment of spray-drying.

Two antitubercular drugs were associated to the microparticles, INH and RFB. These were incorporated in the dispersion at different stages, depending on their solubility in water. INH is a hydrophilic drug. It was first triturated in a porcelain mortar, then weighed in a test tube and solubilised with purified water under mild stirring for 10 min. Afterwards, the dissolution was slowly added to the LBG polymeric dispersion (2%, *w*/*v*), prepared as indicated above, which meanwhile was under stirring overnight. The LBG:INH dispersion was kept under stirring another 90 min until spray-drying (Figure 11, routes A and B). The amount of drug incorporated in the formulation corresponded to an LBG:INH mass ratio of 10:1. RFB is a lipophilic drug and, thus, a solid dispersion of LBG and RFB was prepared prior to the dissolution in order to facilitate the process. The mixture of the two solid materials was performed by geometric dilution and trituration took place in a glass mortar. After grinding, the same procedure used to prepare the LBG dispersion (without drug) was followed (Figure 11, route C). RFB was associated to LBG in amounts leading to theoretical LBG:RFB mass ratios of 10:1, 10:0.5 and 10:0.2.

Polymeric dispersions (LBG) with or without drugs were spray-dried using a Büchi B-290 laboratory mini spray-dryer (Büchi Labortechnik AG, Flawil, Switzerland) equipped with a high performance cyclone. The operating parameters were optimized as follows: inlet temperature: 160 ± 2 °C; aspirator setting: 85%; feed rate: 0.8 ± 0.1 mL/min; and spray flow rate: 473 L/h. These conditions resulted in outlet temperature of 102 ± 1 °C. After spray-drying, microparticles were collected, placed in a dark flask and stored inside a desiccator until further use.

The spray-drying yield was calculated by gravimetry, comparing the total amount of solids initially with the resultant weight of microspheres after spray-drying (*n* = 3).

### 3.4. Characterisation of Microparticles

The surface morphology of produced microparticles was characterised by field emission scanning electron microscopy (FESEM; FESEM Ultra Plus, Zeiss, Jena, Germany). Dry powders were placed onto metal plates and 5 nm thick iridium film was sputter-coated (model Q150T S/E/ES, Quorum Technologies, Lewes, UK) on the samples before viewing.

Microparticle size was estimated as the Feret’s diameter and was directly determined by optical microscopy (Microscope TR 500, VWR International, Leuven, Belgium) from the manual measurement of 300 microparticles (*n* = 3).

Real density (g/cm^3^) was determined using a Helium Pycnometer (Micromeritics AccuPyc 1330, Aachen, Germany) (*n* = 3). Bulk and tap densities (g/cm^3^) were determined using a tap density tester (Densipro 250410, Deyman, Santiago de Compostela, Spain), by measuring the volume of a known weight of powder before and after tapping, respectively (*n* = 3). The determination of tap density involved tapping the sample until no further reduction of powder volume was observed (which corresponded to an average of 180 taps).

The aerodynamic diameter (D_aer_) was determined theoretically and is defined as the diameter of a sphere of unit density that has the same terminal settling velocity as the particle under consideration. It was calculated based on the following equation:

(1) $$D_{aer}=D_{g\;}\sqrt{\frac{\rho_{real}}{\rho_{0\;}\lambda }}$$

where ρ_0_ = 1 g/cm^3^, D_g_ corresponds to geometric diameter (determined as the Feret’s diameter (μm)), ρ_real_ is the real density of microparticles in the same unit as ρ_0_ (g/cm^3^), and λ is the dynamic shape factor of the particle [26,27,56].

### 3.5. Determination of Drug Association

A determined amount of drug-loaded microparticles was incubated with HCl 0.1 M, under magnetic stirring for 60 min, which ensures complete dissolution of the carriers. Samples were then centrifuged (8000 rpm, 30 min; 5810 R, Eppendorf, Hamburg, Germany) and filtered (0.45 μm) before quantification by UV-Vis spectrophotometry (Pharmaspec UV-1700, Shimadazu, Kyoto, Japan) at 265.5 nm and 500 nm for INH and RFB, respectively. A calibration curve was performed using the medium of dissolution of unloaded microparticles, centrifuged and filtered as described for drug samples. A screening of the matrix material (LBG) revealed no interference at the selected wavelengths. Drug association efficiency and microparticle loading capacity were estimated (*n* = 3).

### 3.6. Crystallinity of Dry Powders

The crystallinity of the raw material (LBG), drugs and LBG-based microparticle formulations was evaluated by Powder X-ray diffraction (PXRD). Free drugs were spray-dried in absence of any other excipient for this effect. The samples were analysed in an X’Pert Pro diffractometer (PANalitycal, Almelo, The Netherlands) using nickel filtered CuKα radiation with a wavelength of 0.154 nm. An X’Celerator detector was used and the operating conditions were 45 kV and 35 mA. The diffractograms were obtained in reflection mode from 5 to 60° 2θ with a step size of 0.05° and 1500 s per point. 

### 3.7. In Vitro Drug Release

The release assays were conducted in PBS pH 7.4 added of 1% (*v*/*v*) Tween 80^®^. In order to establish the experimental setup, it was necessary to determine the solubility of INH and RFB in the specific medium. The solubility of INH in PBS pH 7.4 is reported in the literature as 274 ± 4.79 mg/mL [57]. In the absence of a value reported in the literature, an experimental determination has defined the maximum solubility of RFB in PBS pH 7.4 + 1% Tween 80^®^ as 0.496 mg/mL. Sink conditions were respected in all cases, as the maximum amount of drug was always below 30% of its maximum solubility, as advised by the European Medicines Agency [58].

A determined amount of microparticles (of each formulation) were incubated individually with the medium, under mild shaking (100 rpm, orbital shaker OS 20, Biosan, Riga, Latvia) at 37 °C. The released drug was quantified at predetermined times by collecting a sample (1 mL) and performing a quantification by UV-Vis spectrophotometry. A calibration curve was performed using the medium resulting from the incubation of unloaded LBG microparticles with the referred medium, followed by centrifugation (8000 rpm, 60 min) and filtration (0.45 μm). Specifically, for LBG:INH, 10 mg of microparticles were incubated in 12 mL of release medium in a test tube. At specific time intervals (5, 10, 15, 20, 30, 60, 90, 120, 240, 360 and 1440 min), samples were collected individually, filtered (0.45 μm), appropriately diluted (1:3) and the absorbance determined (*n* = 3). For LBG:RFB, 20 mg of microparticles were suspended in 10 mL of release medium. At specific time intervals (10, 20, 30, 60, 90, 120, 180 and 240 min), the samples were collected, centrifuged (16,000× *g*, 15 min, Heraeus Fresco 17 Centrifuge, ThermoScientific, Waltham, MA, USA), filtered (0.45 μm), and the absorbance determined (*n* = 3).

### 3.8. In Vitro Biocompatibility Study

#### 3.8.1. Evaluation of Metabolic Activity

A549 cells were seeded at a density of 1 × 10^4^ cells/well on 96-well plates (Orange Scientific, Braine-l'Alleud, Belgium), in 100 μL of complete DMEM. Cells were incubated for 24 h at 37 °C in 5% CO_2_ atmosphere before use. THP-1 cells were differentiated with PMA to obtain the macrophage-phenotype before the experiments, according to the procedure described above (Section 3.2), with the necessary adaptations. For this assay, THP-1 cells were seeded on 96-well plates (0.035 × 10^6^ cells/well) in 100 μL of RPMI supplemented with 50 nM of PMA and incubated for 48 h at 37 °C in 5% CO_2_ atmosphere. After that time, CCM was renewed for other 24 h, before the experiments.

RFB was pre-solubilised in DMSO at a concentration of 20 mg/mL, being subsequently diluted to the desired concentrations with CCM. CCM and SDS at 2% (*w*/*v*) were used as negative and positive control of cell death, respectively. All test substances were solubilised/dispersed in pre-warmed CCM without FBS, and exposed to both cell lines for 3 and 24 h.

MTT solution (0.5 mg/mL in PBS, pH 7.4) was added after the exposure time (in A549 cells samples were previously removed; in THP-1 cells no removal was applied) and incubated for 2 h, after which formazan crystals were solubilised with DMSO (A549 cells) or 10% SDS in a 1:1 mixture of DMF:water (THP-1 cells) and the absorbance measured by spectrophotometry (Infinite M200, Tecan, Grödig, Austria). The viability of untreated cells was assumed to correspond to 100% of cell viability, and viability of treated cells was compared to this control. The assay was replicated at least three times, each with six replicates.

#### 3.8.2. Determination of Cell Membrane Integrity

Cells were cultured in 96-well plates in the conditions described before for the MTT assay. Upon exposure, cell culture supernatants were collected, centrifuged (16,000× *g*, 5 min, 4 °C) and processed using a commercial kit. Absorbances were measured by spectrophotometry (Infinite M200, Tecan, Austria) at a wavelength of 490 nm (background correction at 690 nm).

A negative control of LDH release was performed incubating cells with CCM only and a positive control corresponded to the lysis solution. Released LDH (%) upon incubation with each sample was determined by comparison with the 100% of the negative control. All measurements were performed in triplicate.

### 3.9. Preliminary Evaluation of Macrophage Ability to Uptake LBG Microparticles

NR8383 cells were seeded (1.0 × 10^6^ cells per well) in 6-well plates for adhered cells, with 2 mL of Ham’s F12 medium. This procedure was performed 24 h before the test to ensure the adhesion of 50% to 75% of the original population.

The evaluation of microparticle uptake by macrophages was performed by flow cytometry (FacScalibur cell analyser, BD Biosciences, Erembodegem, Belgium), which required the exposure to fluorescently-labelled LBG microparticles. These were prepared by dispersing LBG (1.0 g) in a 10^−4^ M HCl solution (pH ~ 4) in order to obtain a final LBG concentration of 1% (*w*/*v*). The same treatment described for microparticle preparation (Section 3.3) was applied. Fluorescein (45.5 mg) was solubilised in 15 mL of ethanol 96% (*v*/*v*) and added to the previously formed LBG dispersion. The added amount of fluorescein was calculated to represent approximately 2% of the number of hexose units in LBG. EDAC (34.79 mg) was dissolved in 15 mL of milli-Q water and added to the dispersion. This was kept under stirring for 72 h, protected from light and then dialysed (2000 Da *M_w_* cut off) against water. The resulting suspension was frozen and freeze-dried (FreeZone Benchtop Freeze Dry System, Labconco, Kansas City, MO, USA). The fluorescently-labelled LBG was stored in a desiccator until further use, under light protection. Fluorescent microparticles were produced according to the same conditions reported in Section 3.3 for unloaded LBG microparticles.

Two doses of microparticles, 220 and 50 μg/cm^2^, were tested. Fluorescently-labelled microparticles were aerosolised onto the macrophage layer using a Dry powder Insufflator™ (Model DP-4, Penn-Century™, Wyndmoor, PA, USA) and incubation allowed for 2 h at 37 °C, without CCM (only a residual amount of medium was kept to ensure the hydration of cell surface). The phagocytic process was stopped by the addition of a cold solution of PBS.3% FBS (5 mL, two applications). Cells were scraped and centrifuged (1500 rpm, 2 min, room temperature, centrifuge MPW-223e, MedInstruments, Warsow, Poland) in 2 mL of PBS.3% FBS. The cycle of resuspension in PBS.3% FBS and centrifugation was repeated thrice. At the end, cells were re-suspended in 1 mL of PBS.3% FBS, transferred to cytometry tubes (BD Biosciences) and maintained at 4 °C until the analysis.

In flow cytometry, FSC-H and SSC-H channels were used, respectively, to measure size and granularity of cells, while side scatter light was used to identify cell viable population. The amount of cells exhibiting a fluorescent signal was considered to have phagocytosed microparticles. The assay for each dose was replicated at least three times.

### 3.10. Statistical Analysis

The student *t*-test and the one-way analysis of variance (ANOVA) with the pairwise multiple comparison procedures (Holm-Sidak method) were performed to compare two or multiple groups. For the analysis of results of *in vitro* release assay, a two-way ANOVA with Bonferroni’s method for multiple comparison test was used. All analysis were run using the GraphPad Prism (version 6.07, GraphPad Software, La Jolla, CA, USA) and differences were considered to be significant at a level of *p* < 0.05.

## 4. Conclusions

This work proposes an alternative inhalable therapy for pulmonary tuberculosis, based on a rather unexplored polysaccharide, LBG. INH and RFB, two first-line antitubercular drugs, were efficiently associated (association efficiencies >82%) to LBG microparticles produced by spray-drying. Microparticles were tailored to evidence theoretically suitable properties (aerodynamic diameters between 1.15 and 1.67 μm) for lung delivery with the objective of reaching the alveolar zone, where macrophages reside. Importantly, LBG microparticles evidenced strong ability to be captured (percentage of phagocytosis >94%) by two different macrophage-like cells (macrophage-differentiated human THP-1 cells and rat alveolar macrophages NR8383). While INH-loaded microparticles demonstrated to be devoid of cytotoxicity towards epithelial A549 cells and macrophage-differentiated THP-1 cells at concentrations up to 1 mg/mL and exposure times up to 24 h, RFB-loaded microparticles revealed a considerable cytotoxic effect from 0.5 mg/mL on. The work described herein also pioneered the spray-drying of LBG, which is a valuable contribution to the state of the art. Drug-loaded microparticles were found not to display a crystalline pattern, but this is not expected to compromise the overall potential identified for the proposed system regarding tuberculosis therapy. Nevertheless, unveiling the effect of a long-term administration of LBG microparticles *in vivo* is a very relevant aspect to address before establishing the real potential of the approach.

## Figures and Tables

**Table 1:** Spray-drying production yields and microparticle Feret’s and aerodynamic diameters (mean ± SD, *n* = 3).

Formulation	LBG/Drug (*w*/*w*)	Production Yield (%)	Diameter (μm)		
			Feret’s	Aerodynamic, λ = 1	Aerodynamic, λ = 2
Unloaded LBG	10:0	70.1 ± 4.1	1.35 ± 0.73	1.59 ± 0.06	1.12 ± 0.04
LBG:INH	10:1	66.0 ± 5.8	1.50 ± 0.80	1.83 ± 0.21	1.30 ± 0.16
LBG:RFB	10:0.2	61.5 ± 0.7	1.26 ± 0.63	1.54 ± 0.21	1.09 ± 0.16
	10:0.5	67.0 ± 2.8	1.10 ± 0.56	1.27 ± 0.01	0.89 ± 0.01
	10:1	70.1 ± 4.8	1.50 ± 0.86	1.78 ± 0.03	1.26 ± 0.03

INH: Isoniazid; LBG: Locust Bean Gum; RFB: Rifabutin; λ: shape factor (1: spherical shape; 2: irregular shape).

**Table 2:** Microparticle real, bulk and tap densities (mean ± SD, *n* = 3).

Formulation	LBG/Drug (*w*/*w*)	Density (g/cm^3^)		
		Real	Bulk	Tap
Unloaded LBG	10:0	1.39 ± 0.01	0.24 ± 0.06	0.37 ± 0.08
LBG:INH	10:1	1.41 ±0.02	0.24 ± 0.01	0.36 ± 0.00
LBG:RFB	10:0.2	1.41 ± 0.03	0.20 ± 0.01	0.32 ± 0.05
	10:0.5	1.33 ± 0.03	0.15 ± 0.04	0.25 ± 0.07
	10:1	1.39 ± 0.02	0.14 ± 0.02	0.25 ± 0.02

INH: Isoniazid; LBG: Locust Bean Gum; RFB: Rifabutin.

**Table 3:** Drug association efficiency and microparticle loading capacity determined for LBG microparticles (mean ± SD, *n* = 3).

Formulation	LBG/Drug (*w*/*w*)	Association Efficiency (%)	Loading Capacity (%)
LBG:INH	10:1	88.8 ± 1.5	8.8 ± 0.1
LBG:RFB	10:0.2	92.4 ± 6.0	1.8 ± 0.1
	10:0.5	86.3 ± 3.0	4.1 ± 0.1
	10:1	102.8 ± 3.8	10.3 ± 0.4

INH: isoniazid, LBG: locust bean gum, RFB: rifabutin.

## Supplementary Materials

Supplementary materials can be accessed at: http://www.mdpi.com/1420-3049/21/6/702/s1.
