# Supplementary material for: Inhalable Antitubercular Therapy Mediated by Locust Bean Gum Microparticles
Source: Molecules. 2016 May 28;21(6):702. doi: 10.3390/molecules21060702 (PMC6273308; doi:10.3390/molecules21060702)
Supplement: Supplementary file 1 [file molecules-21-00702-s001.pdf]

Supplementary Materials: Inhalable Antitubercular Therapy Mediated by Locust Bean Gum Microparticles

Ana D Alves, Joana S Cavaco, Filipa Guerreiro, João P Lourenço, Ana M Rosa da Costa and Ana Grenha

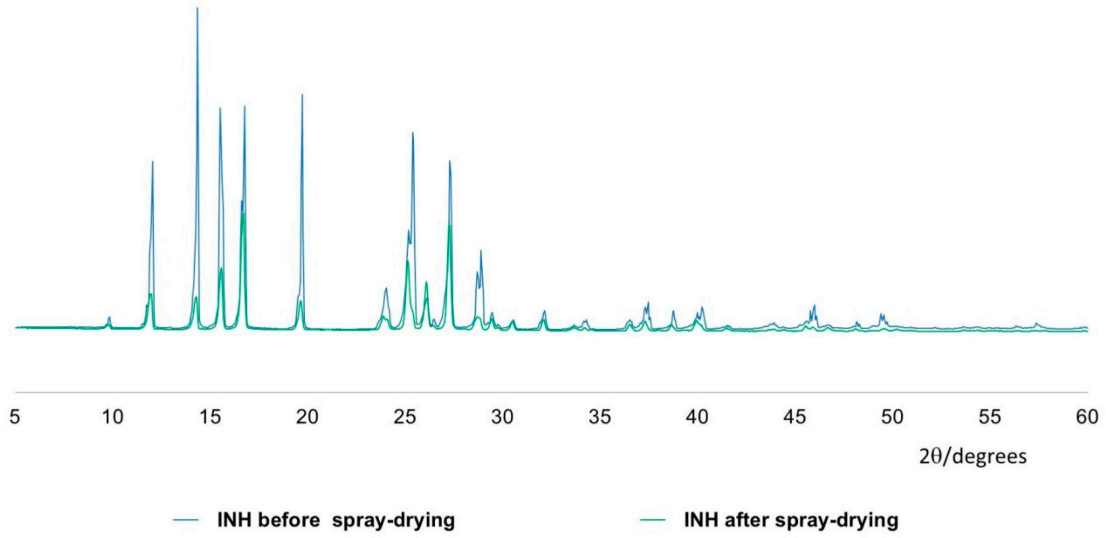

Figure S1. XRD spectra of isoniazid (INH) before and after spray-drying.

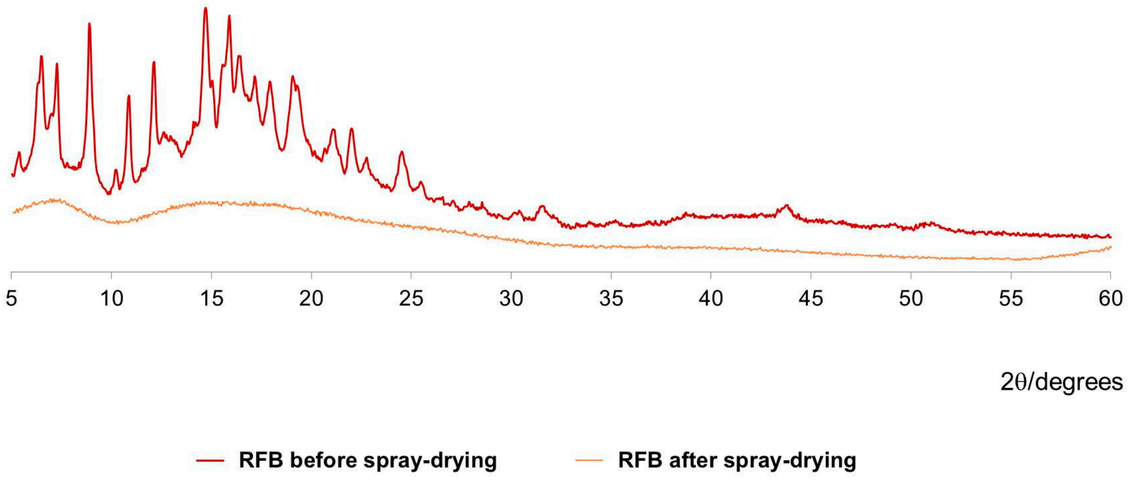

Figure S2. XRD spectra of rifabutin (RFB) before and after spray-drying.

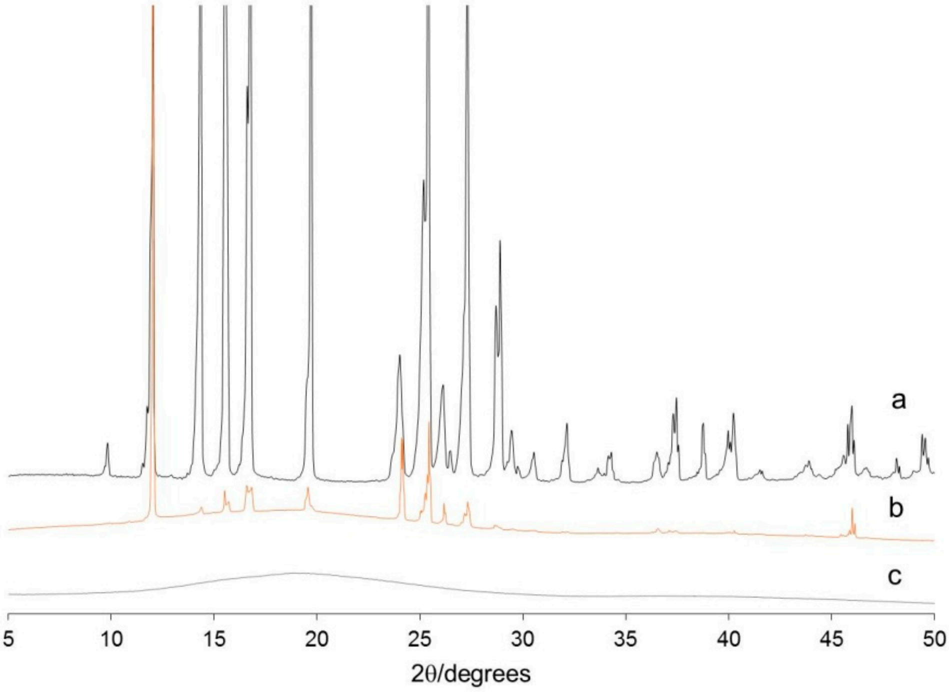

Figure S3. XRD spectra of (**a**) isoniazid (INH) in pure state; (**b**) physical mixture of locust bean gum (LBG) and isoniazid in mass ratio of 10/1; and (**c**) LBG.INH microparticles.

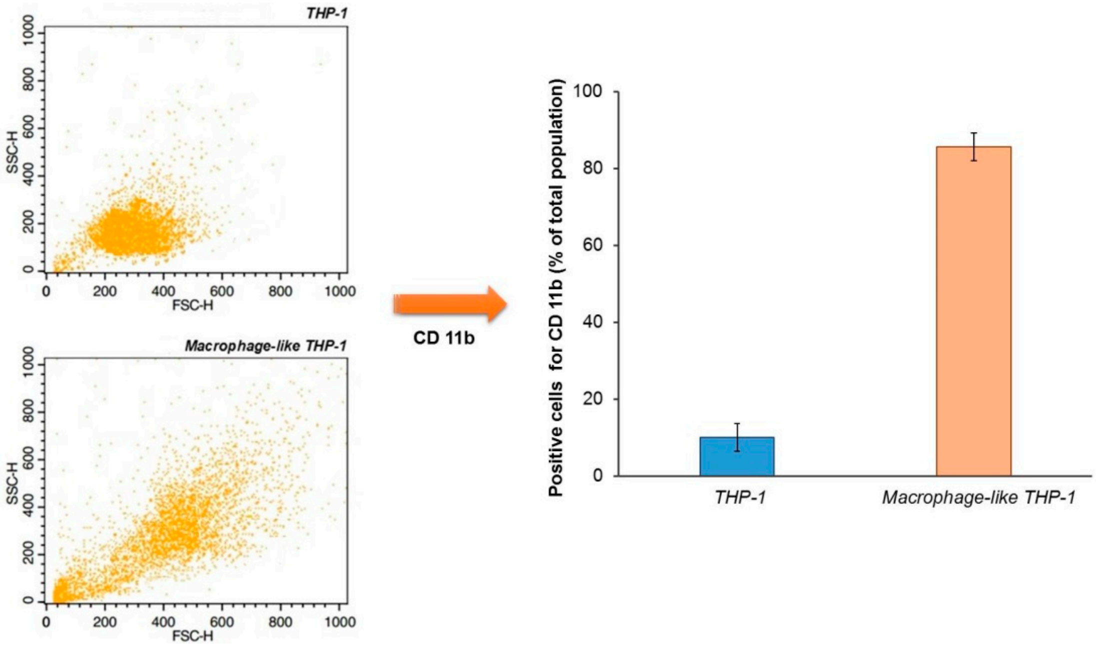

Figure S4. Immunocytochemical characterization of THP-1 cells and macrophage-differentiated THP-1 cells. Evaluation and comparison between size (axis FSCH) and structure/complexity (axis SSCH) of THP-1 and macrophage-like THP-1 cells. Macrophage-like THP-1 cells were differentiated by 48 h incubation with 50 nM of PMA. Marker anti-CD 11b labels the CR3 receptor present only in membrane of THP-1 differentiated cells. Results are expressed as mean ± SEM.

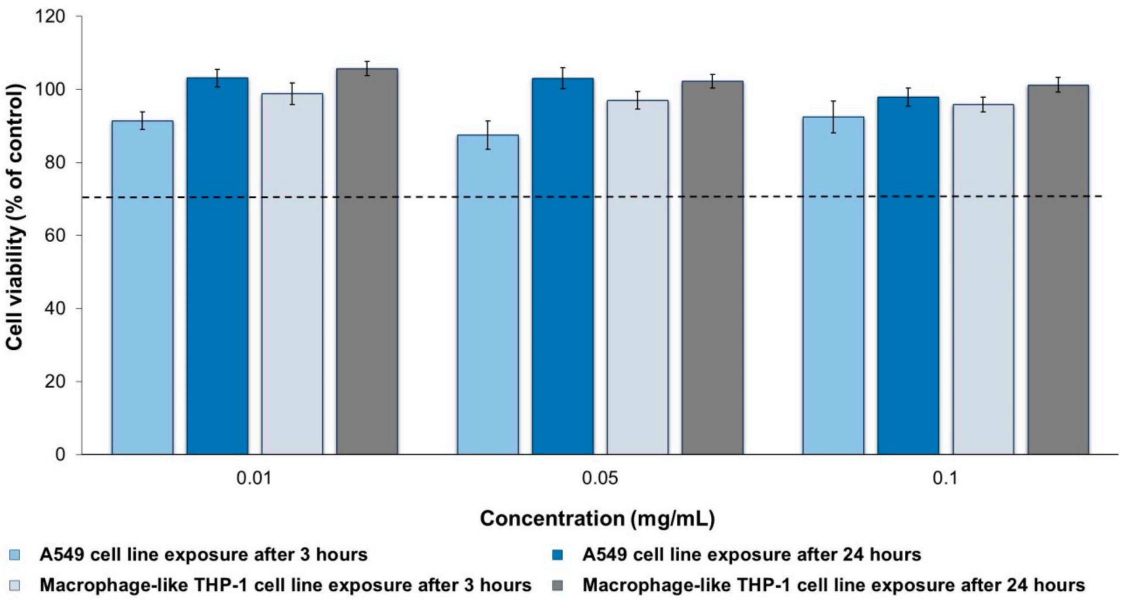

Figure S5. A549 (**blue**) and macrophage-differentiated THP-1 (**grey**) cell viabilities after 3 h (**lighter** colour) and 24 h (**darker** colour) of exposure to isoniazid. Results are expressed as mean ± SEM (n = 3, six replicates per experiment at each concentration). Dashed line represents 70% cell viability.

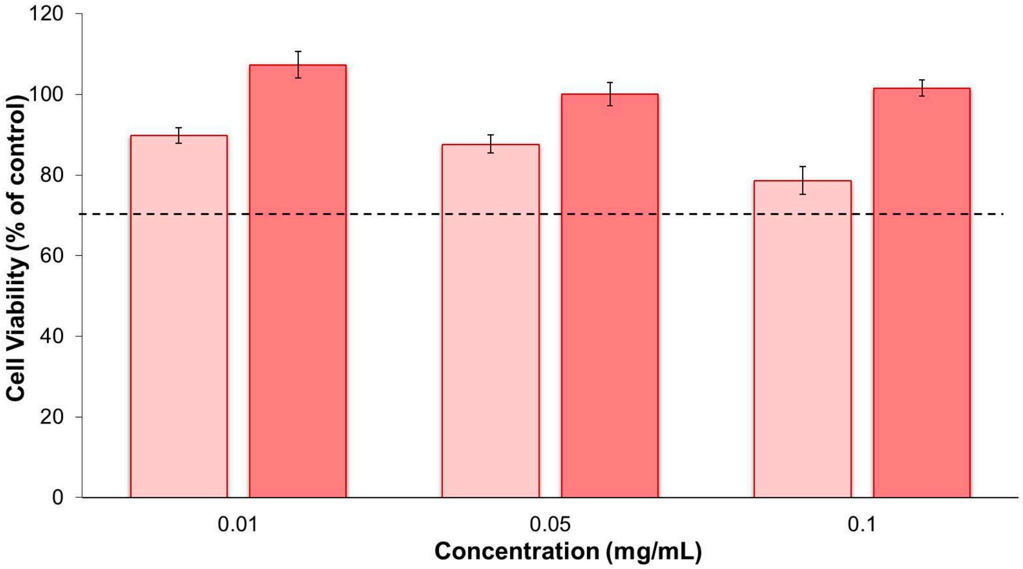

Figure S6. A549 (**lighter** colour) and macrophage-differentiated THP-1 (**darker** colour) cell viabilities after 3 h of exposure to rifabutin. Results are expressed as mean ± SEM (n = 3, six replicates per experiment at each concentration). Dashed line represents 70% cell viability.

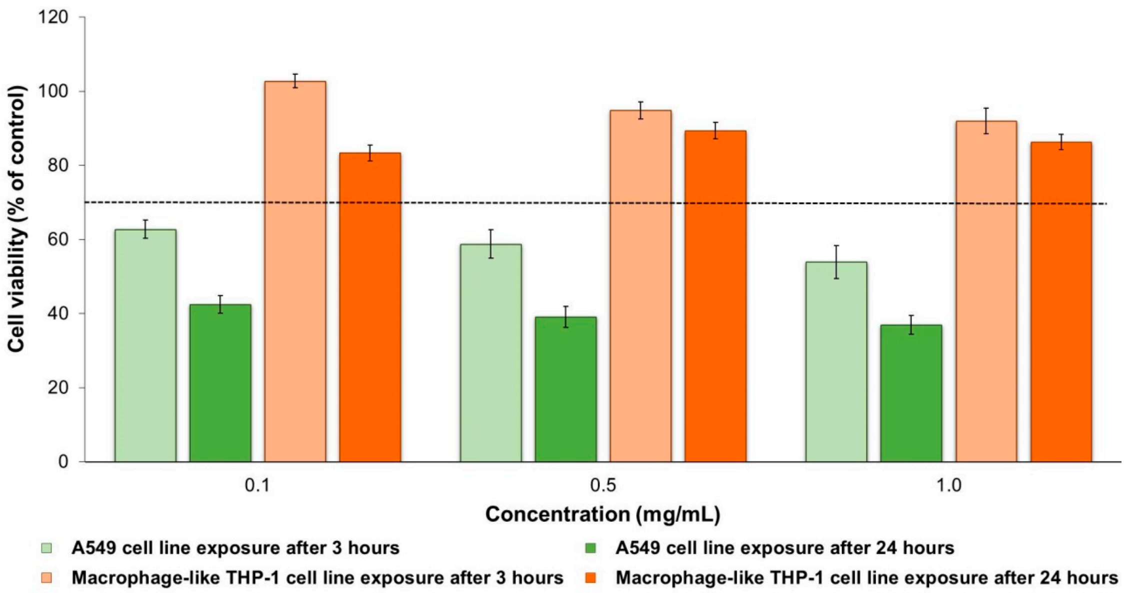

Figure S7. A549 (**green**) and macrophage-differentiated THP-1 (**orange**) cell viabilities after 3 h (**lighter** colour) and 24 h (**darker** colour) of exposure to raw material locust bean gum (LBG). Results are expressed as mean ± SEM (n = 3, six replicates per experiment at each concentration). Dashed line represents 70% cell viability.

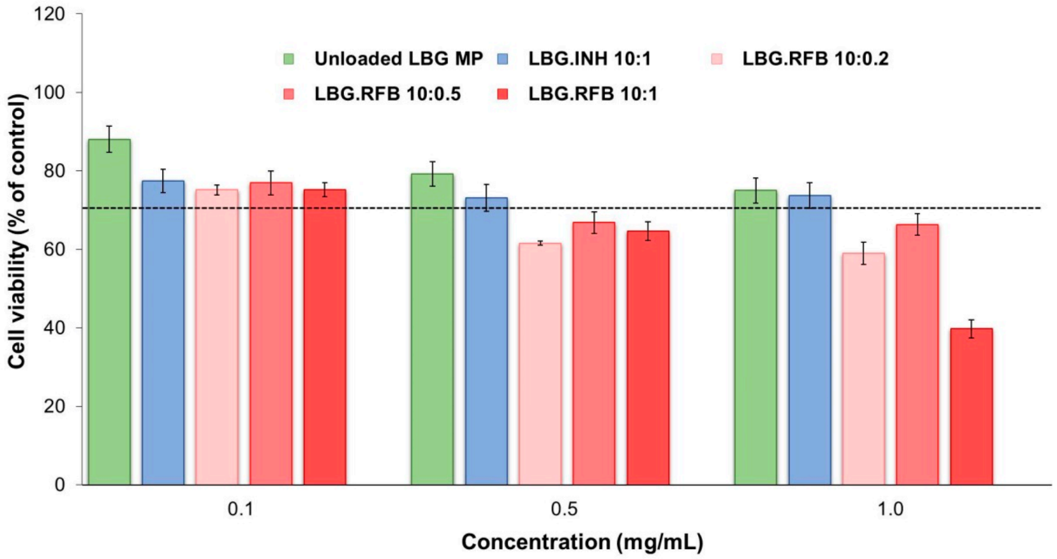

Figure S8. A549 cell viabilities after 3 h of exposure to LBG-based microparticle formulations. Results are expressed as mean ± SEM (n = 3, six replicates per experiment at each concentration). Dashed line represents 70% cell viability (INH: isoniazid, LBG: locust bean gum, RFB: rifabutin).

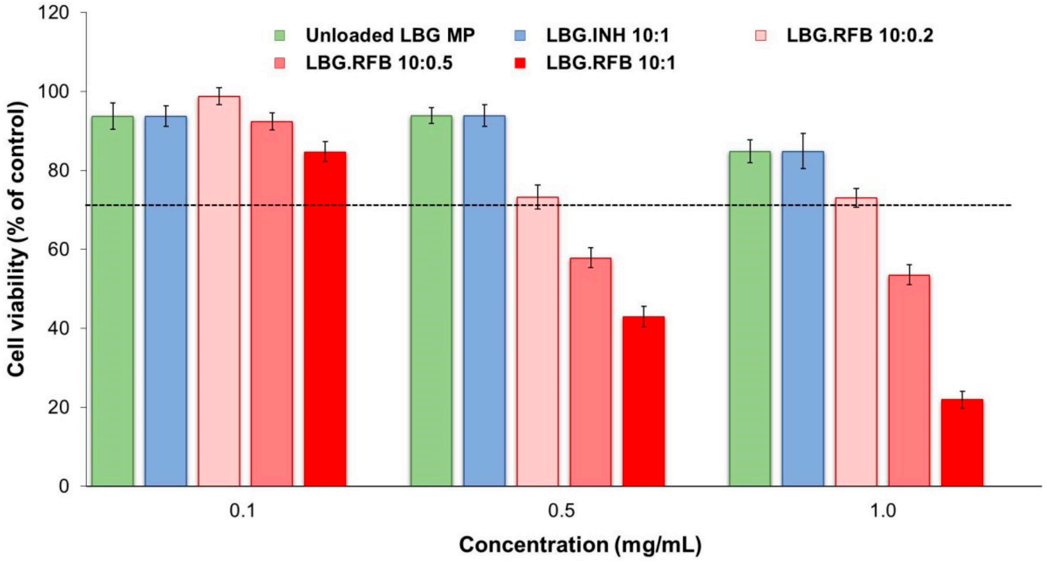

Figure S9. Macrophage-differentiated THP-1 cell viabilities after 3 h of exposure to LBG-based microparticle formulations. Results are expressed as mean ± SEM (n = 3, six replicates per experiment at each concentration). Dashed line represents 70% cell viability (INH: isoniazid, LBG: locust bean gum, RFB: rifabutin).

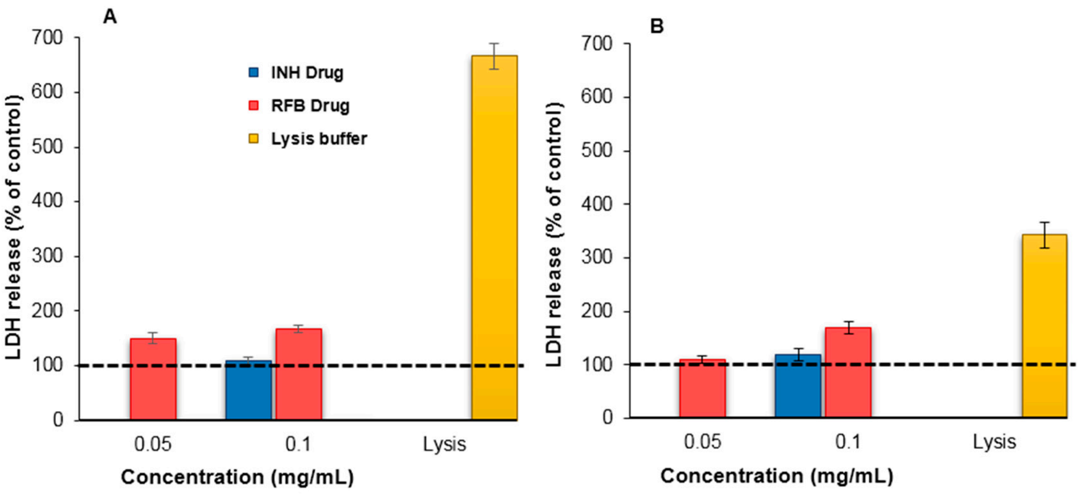

Figure S10. LDH released from A549 cells (**A**) and macrophage-like THP-1 cells (**B**) after 24 h exposure to free drugs (isoniazid: INH, rifabutin: RFB). Amount of LDH released from cells incubated with cell culture medium is assumed as 100% (dashed line). Results are expressed as mean ± SEM (n = 3, six replicates per experiment at each concentration).

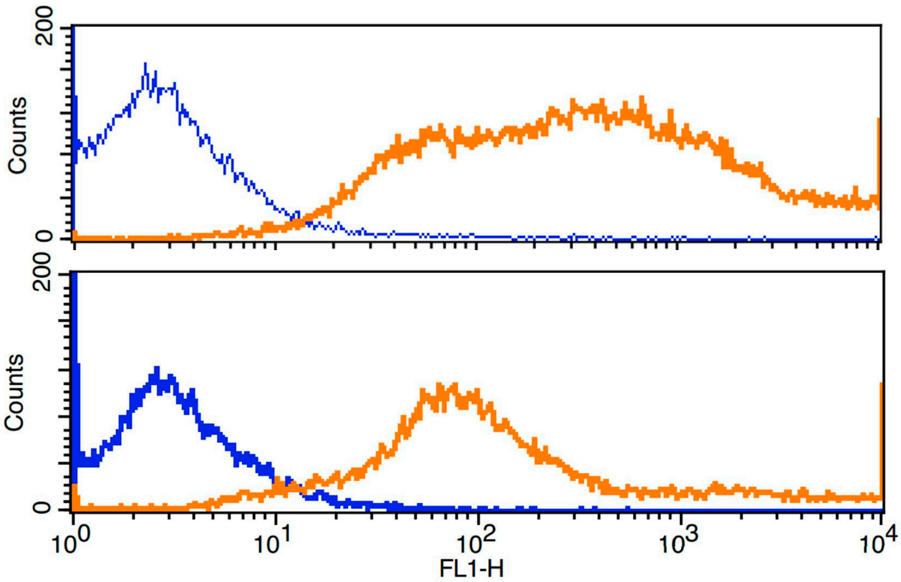

Figure S11. Fluorescent signal of macrophage-differentiated THP-1 (**upper** panel) and NR8383 cells (**lower** panel) upon 2 h exposure to 50 µg/cm² of fluorescently labelled LBG microparticles (**orange** line) and unexposed cells, incubated with cell culture medium (**blue** line).
